# Between health care and social security – psychiatric patients and the disability pension system in Norway and Russia

**DOI:** 10.1186/1472-6963-7-128

**Published:** 2007-08-16

**Authors:** Grigory Rezvyy, Walter Schönfelder, Terje Øiesvold, Reidun Olstad, Georges Midré

**Affiliations:** 1 Nordland Hospital, Psychiatric department, N-8092, Bodø, Norway; 2 Institute of sociology, Faculty of social science, University of Tromsø, Norway; 3 Department of clinical psychiatry, Institute of clinical medicine, University of Tromsø, Norway; 4 University Hospital of Northern Norway, Tromsø, Norway

## Abstract

**Background:**

The official statistics of persons with mental disorders who are granted disability pension (DP) in Russia and Norway indicate large differences between the countries.

**Methods:**

This qualitative explorative hypothesis-generating study is based on text analysis of the laws, regulations and guidelines, and qualitative interviews of informants representing all the organisational elements of the DP systems in both countries.

**Results:**

The DP application process is initiated much later in Norway than in Russia, where a 3 year occupational rehabilitation and adequate treatment is mandatory before DP is granted. In Russia, two instances are responsible for preparing of the medical certification for DP, a patients medical doctor (PD) and a clinical expert commission (CEC) while there is one in Norway (PD). In Russia, the Bureau of Medical-Social Expertise is responsible for evaluation and granting of DP. In Norway, the local social insurance offices (SIO) are responsible for the DP application. Decisions are taken collectively in Russia, while the Norwegian PD and SIO officer often take decisions alone. In Russia, the medical criterion is the decisive one, while rehabilitation and treatment criteria are given priority in Norway. The size of the DP in Norway is enough to cover of subsistences expenditure, while the Russian DP is less than the level required for minimum subsistence.

**Conclusion:**

There were noteworthy differences in the time frame, organisation model and process leading to a DP in the two countries. These differences may explain why so few patients with less severe mental disorders receive a DP in Russia. This fact, in combination with the size of the DP, may hamper reforms of the mental health care system in Russia.

## Background

The Mental Health Declaration for Europe states that one of the most important tasks of the World Health Organisation (WHO) is the promotion of mental health in all European Countries [1]. This task is achievable if there is coordination across countries and an awareness of each country's existing needs and resources. It is also stressed that encouraging of "the development of specialized expertise within the mental health workforce, to address the specific needs of groups such as ... those with long-term and severe mental health problems" is, among others, the responsibility of WHO [2].

For long-term disorders, a disability pension (DP) is considered to be an important aspect of public support. Many European countries have noted a dramatic increase in number of persons receiving DP since 1980 [3] especially for musculoskeletal and mental disorders. Mental disorders are of special concern because of their early debut and increasing proportion among DP recipients. At the same time, both illness certification and the DP systems are considered to be far from ideal, especially when it concerns complex, chronic diseases which lack so called "objective findings" [4,5].

As most of the previous studies have been carried out in western countries, it would be of particular interest to compare the situation in a western country like Norway with the situation in Russia, a country with a very different historic development – both the mental health care system as well as the social security system. Describing these differences in a systematic way, using internationally validated instruments developed for cross-cultural research, would give new knowledge especially on how cultural factors, different professional traditions and history influence structure and function of the mental health care and social support systems. This new knowledge could give important input into the process of further developing the mental health care and social support systems in the two countries.

A previous comparative study of the mental health care system has shown large differences between the two countries [6]. There is a greater number of beds per 100000 inhabitants for long term patients with severe chronic mental disorders in Archangelsk County (Oblast) than in Northern Norway. Although many of the long term patients in Archangelsk could be discharged if only their mental disorder were taken into account, the general impression is that the clinicians have assumed that these patients could not cope outside the hospital. The lack of locally based services such as sheltered housing, community based psychiatric services and the difficult economical situation for patients outside the hospitals are factors that make the discharge of patients challenging. Even if the patients, after having been discharged from the hospital, get a chance to be placed in a sheltered home called "internats for chronic psychiatric patients", this is not necessary a guaranty for improvement of the situation, as few individuals ever return from internats to the community [7].

Existing statistical information indicates that the total number of disability pensioners per population (2004) is comparable in Norway (6, 75%) and Russia (7, 36%) [8,9]. When it comes to DP on the basis of a mental disorder, the numbers of new disability pensions are of special interest, as this reflects current practices within the DP system. In Norway, a psychiatric diagnosis was the primary diagnosis of 21.5% of all new DP given in 2004, compared to 3.0% in Russia [10]. Schizophrenia was the primary diagnosis of 2% of all new DP given in Norway in 2004, which was approximately 10% of those given DP with psychiatric disorders. In Russia, 1.1% of all new DP were given to persons with schizophrenia, which was approximately 30% of those given DP on the basis of a mental disorder. In Norway, approximately 18.2% of all new DP were given on the basis of affective disorders, neurosis and personality disorders in 2004. In a study from Moscow in 2001, a primary diagnosis of schizophrenia was given to 65% of all new DP with psychiatric disorders, while neurosis and personality disorders were the primary diagnosis of 1.9% of all new DP with psychiatric disorders [11].

These figures indicate large differences between the two countries as to how the DP system is used in connection to mental disorders. However, the reasons for this difference are unclear. The Norwegian DP model is well described in international journals [3,12-16] while descriptions and analyses of the Russian DP are scarce and often only published in Russian journals [17,18].

The procedure of DP certification has several steps: the initiation of the process, the patient evaluation and the final decision making process. In case of mental disorders, the initiation of the process is complicated by the fact that patients with severe mental illness often lack insight into their disorder and may not accept that they are ill and/or in need of a DP. The evaluation includes evaluation of both the patient's mental status, psychiatric diagnosis as well as functional and social status. One crucial issue is to decide if the patient fulfils the legal criteria for a DP. Differences between countries might result from different legislation, especially when it comes to criteria for granting DP. Differences in legislation could reflect differences between the countries as to how the concept "disability" is understood in the society as it is likely influenced by cultural factors [19,20]. Of particular relevance is how the medical criteria (diagnosis, treatment/rehabilitation outcome, and prognosis) and functional/social criteria are balanced. In addition to what is in the legislation and regulations, how these criteria are interpreted and used in practice is crucial. The professional background of the persons involved and the way the decision-making process is organised may influence the final result of the process.

The goal of the study was to describe differences and similarities in the DP system between Norway and Russia, using severe mental disorders as an example. The sub-goals were to describe and compare:

1. The legislation for granting DP in the two countries with specific focus upon the criteria for granting DP.

2. Procedures for granting of the DP with special focus upon:

- The institutions and persons involved in the process.

- The practical use of the legal criteria for granting DP.

3. The economic magnitude of the DP that was granted.

## Methods

The study includes a review of available statistical data and an analysis of the laws, regulations and guidelines in both countries with a special focus upon the criteria for granting DP. The criteria were categorised into the following themes: medical criteria, functional criteria and criteria connected to time aspects/duration of illness. Relevant textbooks and literature recommended for use by juridical and medical personal were analysed. The material was used to give a general description of the organisation of the DP systems, application processes and organisations involved.

To study the actual procedures for DP – granting in practice, data were collected by qualitative interviews with a total of 10 participants in Norway and 14 participants in Russia [21]. Participants were recruited through face to face or telephone contact. Data was collected in Archangelsk, Russia and two cities in Northern Norway – Tromsø and Bodø during 2005/2006.

As a result of the differences in the DP systems in the countries, the number and positions of the participants varied. In Russia, the participants represented a mental hospital (two psychiatrists and two social workers), an out-patient psychiatric clinic (three psychiatrists), a rehabilitation centre (two social workers), the pension fund (two officers) and the Psychiatric Bureau of Medical-Social Expertise (PBMSE) (three psychiatrists). In Norway, the participants were one general practitioner (GP), two psychiatrists and one clinical social worker working at a mental hospital, one officer representing local social insurance office (SIO), one county SIO officer, one rehabilitation adviser and three social workers representing the social security office (SSO).

In Russia, the interviews were conducted by three of the authors: a professor in sociology (GM), research fellow in sociology (WS) and a Russian psychiatrist, currently working in Norway as research fellow (GR). A professional interpreter was used for the non-Russian members of the research team. In Norway, the interviews were conducted by GR alone. The interviews were conducted by GR in the native tongue of the interviewees in both countries with simultaneous translation to the rest of the interviewer-group in Russia. All interviews were recorded and transcribed for subsequent analysis.

The semi-structured interviews were 1/2–1 hour in duration. Each began with an introduction by GR who presented the interviewer-group and clarified the focus of the study. A case-vignette describing a patient suffering from chronic schizophrenia was used as a central element of the interview. Participants were encouraged to read the case and talk freely following the interview topics. A brief interview-guide was used to lead the discussion towards following topics: general comments to the case, criteria, the process of applying and granting of DP and magnitude of the pension.

The interviews were coded and analysed with the N'Vivo computer program for qualitative data analysis.

### The case-vignette

A male patient, 35 years old, has been admitted at the mental hospital four times during the last 3 years with a diagnosis of Schizophrenia. As usual, the patient has been discharged from the hospital following some improvement. However, he has never been totally rid of his delusions despite adequate medical treatment. He is an educated carpenter but lost his job several years ago as a result of his psychiatric problems. He was helped to find another job and has tried to work several times, but he has not been able to manage the work. He is divorced and lives together with his mother. However, his mother does not want to have him in her house any more, after that he has become increasingly psychotic and rude with her. He has not had contact with his ex-wife in the last 2 years.

## Results

### 1. The legislation for granting DP in the two countries with focus upon criteria for DP granting

In Norway: According to the National Insurance Act (NIA) [22] the purpose of the disability pension is to secure income for persons of employment age with a reduced earning ability due to a lasting illness related reduction in working capabilities (§ 12-1).

#### Rehabilitation/treatment criteria

The first decisive criterion for granting DP in Norway is that the patient has gone through a comprehensive occupational rehabilitation and treatment program before he may apply for DP (§ 12-5) [23]. This criterion can be set aside if there are obvious reasons to evaluate that this criterion is not appropriate. It must be documented that the patient has been involved in occupational rehabilitation simultaneously with relevant treatment (minimum 3 years), and has not achieved a satisfactory result. If this criterion is fulfilled, the medical criterion will be evaluated. Of applications for DP for mental disorders, 23% and 32% were rejected for women and men, respectively in 2002. Of the applicants, 13.2% were rejected as a result of a lack of adequate treatment, while 49% were rejected due to a lack of occupational rehabilitation. In 2000, the percentage of applications rejected due to lack of occupational rehabilitation was 20.4%, which demonstrate the current importance of the treatment rehabilitation criteria [24].

#### Medical criteria

The illness should be defined as "a disease which is scientific-based and generally accepted in medical practice" [25]. Usually, the disease should be included in the currently used International Classification of the Diseases (ICD-10) [26]. However, not all diseases in the ICD-10 provide adequate grounds for granting DP.

#### Functional criteria

The disease should cause lasting functional reduction of earning ability in minimum 50% (§ 12-7). Factors such as age, abilities, education, occupational background and ability to work where the person resides as well as in the rest of the country, should be taken into account.

#### Duration – time aspects

To apply for temporary DP, a patient has to have a disease which has lasted not less than 4 years and will likely persist at least 1 more years. Permanent DP can be granted after a minimum period of 7 years uninterrupted disability.

#### Relationship to other economical support

After the start of an illness, the patient will have the right to paid illness leave for the first 12 months. Subsequently, during the period of treatment and rehabilitation, the patient may receive rehabilitation support for the next 3 years before applying for a DP. In addition to a DP, patients may apply for economic social assistance.

In Russia: In the federal acts, "On labor pensions in the Russian Federation" of 17.12.2001 No 173-Φ 3 [27] and "On the state pension providing system in the Russian Federation" from 15.12.2001, No 166-Φ 3 [28], the definition and understanding of DPs are quite similar in Russia and Norway, with the exception that persons in pension age can also apply for DP.

#### Medical criteria

As in the Norwegian legislation, no specific diagnosis is required for granting of a DP.

#### Functional criteria

A disease resulting in a lasting functional reduction must be the main reason for the reduction in earning ability of at least 50%. Before 1990, the main criterion in DP granting in Russia was permanent reduction of the working capacity. The new Federal law "On social protection of invalids in the Russian Federation" from 24.11.1995, No 181 [29], introduced a revised definition of "invalid" based on "limitations in vital activities" (that can be defined as general functional disability) which express themselves in a permanent reduction of a variety of vital functions. The guidelines for medical experts working with the application of the law suggest that the evaluation of the general functional disability should be carried out through a global concrete analysis of individual's "adaptivity" in 7 different social-psychological domains: movement, self-service, education, working capacity, social capacity, self-criticism and communication. These 7 domains are linked to the definition of the grade of the DP, which in turn is linked to the amount of economic support the person receives (se chapter 3).

#### Treatment/rehabilitation criteria

The main difference from the Norwegian model concerns the requirement for compulsory occupational rehabilitation before the DP application can be accepted. In Russia, a rehabilitation plan for the patient is worked out after the granting of a DP.

#### Duration

If the disease has lasted more than a total of 10 months (or more than 4 contiguous months) with an assumed unfavourable prognosis, the patient should apply for a temporary DP (for 1–2 years for all psychiatric patients). A permanent DP may be granted after 7–8 years of uninterrupted disability.

#### Relationship to other economic support

As in Norway, the patient has the right to paid sick leave for the first ten to twelve months of the illness. There is no specific economic support nor is there an alternative stable public support besides DP.

**Figure 1:**
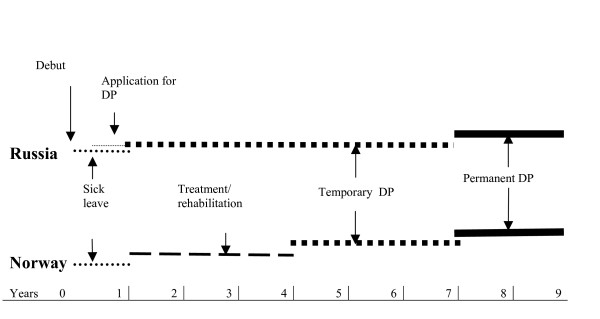
The disability pension (DP) process in Norway and Russia: An overview.

An overview of the economical support system connected to chronic diseases in the two countries is given in figure 1.

### 2. Procedures for granting of the DP

#### The institutions and persons involved in the process

In both countries, the process will usually be initiated when a patient has written and sent an application for a DP. If the patient's medical doctor or another person assumes that the patient is in need of a DP, they can take the initiative to help the patient send an application. However, there were differences between the participants from Russia and Norway as to how much they would press the patient to apply for a DP. The Russian participants were more eager to persuade both the patient and other involved persons (family members, social workers etc.) to apply for a DP.

*If the patient denies applying or does not understand, I will explain to him what it is about, that he is going to get some privileges. I will explain this to his relatives and social workers who are in contact with him. I do not remember any case when we did not manage to persuade a patient to apply*.

(Russian psychiatrist, out-patient psychiatric clinic)

Some of the respondents linked this emphasis on persuasion with the lack of other stable economic solutions beside DP for the patient.

*We have had patients who denied applying for DP and they have no money to live on... We can always find some compromise with patients. If the patient offers a substantive proposal, we can discuss this and find some solution which is satisfactory for all of us*.

(Russian psychiatrist, mental hospital)

The Norwegian participants were more engaged to find another solution in this situation:

I have had such patients who denied applying. In this case I usually respect their decision. He may be afraid to stay disabled for the rest of the life... Even if I actually do not agree with them, I used to respect the decision first of all... We will go as far as possible to meet his wish.

(Norwegian psychiatrist, mental hospital)

In Norway, the local social insurance offices (SIO) are responsible for further processing of the DP application and the SIO officer responsible will ask the patient's doctor (PD) to write a medical certification. The PD is often the local general practitioner (GP) or the psychiatrist treating the patient at that time. Sometimes the SIO officer sends a general inquiry to the local GP and asks the psychiatrist to answer some specific questions. There are different routines concerning this issue in different Norwegian counties.

**Figure 2:**
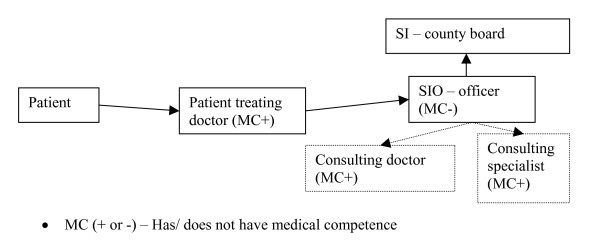
The Norwegian temporary DP process.

The PD is primarily responsible for the medical evaluation as well as providing the first determination as to whether or not the necessary criteria are met. He/she can carry out the evaluation alone, or ask other colleagues to take part. However, there are usually no established routine for such cooperation. The SIO officer, who has more education and additional training, makes the decision when it comes to temporary DP. In the case of permanent DP, the SIO prepares the case for the county SIO board that makes the final decision. If the officer needs to discuss the case with someone with medical competence, he/she can contact a consultant medical officer at the SIO (usually not psychiatrist) (see figure 2).

In Russia, specialized organizations deal with DP. Nationally, the Federal Bureau of Medical-Social Expertise (FBMSE) is organized under the Ministry of Health and Social Development. On the County level, "The Main Bureau of Medical-Social Expertise" (MBMSE) is responsible for the evaluation and granting of DP as well as the preparation and supervision of a plan for rehabilitation for each disabled patient. Branches of the MBMSE usually serve about 70000 inhabitants and handle 1800–2000 applications per year. Most of these branches do not have psychiatrists. However, among the 27 branches of the MBMSE in Archangelsk County, one is a Psychiatric Bureau of Medical-Social Expertise (PBMSE). The PBMSE is responsible for evaluation of all patients with psychiatric diagnoses living in the city of Archangels. The staffs of the PBMSE consists of three psychiatrists certified as experts in social insurance field (one is chief of the bureau), a nurse-registrar, a specialist in rehabilitation (medical doctor), a psychologist and a specialist in social work. However, the three last positions are currently vacant at the PBMSE in Archangelsk County.

**Figure 3:**
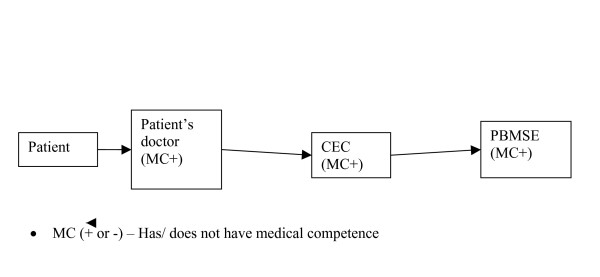
The Russian Model temporary DP process.

At the medical hospital or outpatient clinic where the patient is being cared for, a Clinical-Expert Commission (CEC) consisting of three members; the patient's treating psychiatrist (PD), the psychiatrist in charge of the department and the chief-physician or his/her deputy is established. The PD is responsible for the preparation of the medical and other documentation and the diagnostic work. Other members of the CEC will participate in at least one meeting, hear the report of the PD, speak to the patient and his close relatives, discuss the case and make a decision if an application should be sent as well as sign the medical certification.  Afterwards, the document will be sent to another three psychiatrists at PBMSE – with more competence in evaluation of DP applications. The patients can also apply for DP directly to the Bureau (without CEC reference) if they have enough medical documentation (see figure 3).

In Russia, placing of the disabled psychiatric patients under guardianship is a common practise. Relatives of the patients or representatives of the institution where the patients are admitted can apply for a statement that the patient is incapacitated. This application will be send to the bureau which is responsible for the assessment. However, the declaration of incapacity will be made by a social security court. One month after the decision is made, the chairman of the local municipality has to nominate an official guardian for the patient: a personal one (usually a relative) or juridical one (an institution, if the patient has been admitted for a long time without positive prognosis to be discharged). In all cases, the disability pension can be used only for the patient's needs. There is organized a control commission at the municipalities which is responsible for revision of all guardians. At the institution, pensions for all admitted disabled patients are placed on a special account and are strictly controlled. The director is a head of so called "guardian board" which is responsible for use of the money only for the patient's needs.

In Norway, the declaration of incapacity is also dependant upon a court decision. However, this possibility is very seldom used in Norway.

#### The practical use of the legal criteria for granting DP

The written case presentation was used as an introduction to the discussion about the criteria for granting DP in the interviews. All participants evaluated the case as a typical DP issue and that the necessary criteria were presented. Almost all of the participants in both countries had evaluated similar patients and used their own professional experience in the evaluation of the case-vignette.

##### The diagnostic hierarchy and need for expertise in the diagnostic process

Even if the demand for a diagnostic evaluation was apparent in both the Norwegian and Russian interviews, it was more emphasized in the Russian ones. It was also apparent that the ability to undertake such an evaluation was to a larger extent among the Russians associated with the status as an expert in the field. The following quotation is an example:

*Yes, a diagnosis is the most important factor. And the diagnosis should be made on a high professional level, the so-called expert level: it should include severity of the disease, whether the patient has some mental defects, how much his psychic functioning reduced and so on*.

(Russian psychiatrist, out-patient department)

In contrast, a distinct aspect of the Norwegian interviews was the emphasis of the independent status of the GP as the responsible person for the medical evaluation. This was expressed in the following quotation:

*But here in Tromsø, it is not usual for GP to get outside help in the evaluation of DP cases*.

(Norwegian GP)

Both Russian and Norwegian specialists pointed out that it is easier to get DP for patients with psychotic diagnosis (schizophrenia) than those with a neurosis or personality disorder. However, the Russian psychiatrists had clearer hierarchical preference among diagnoses:

*Yes, if the patient has schizophrenia, it is usual for this kind of patient to be granted a pension, as it is difficult for him to deal with others... So, in my certification, I will argue, on based on the nature of the nature of the disorder.... And with such a diagnosis he will get DP much more easily*. (Russian psychiatrist, mental hospital)

The Russian respondents have argued for diagnostic preference and use both clinical and social-economic criteria, with the importance of the latter being the more critical:

*It would be more difficult to apply for a patient with personality disorder and even more so for patients with a neurosis. In this case, you need to prove high level of social dysfunction: that he has frequently changed employment, many family issues, and many hospitalizations and so on*.

(Russian psychiatrist, mental hospital)

Also in the Norwegian interviews, the different psychiatric diagnoses were considered in a similar hierarchy but not as a reason to delegate the process to a higher level of expertise. In the case of patients with non psychotic disorders, the diagnostic process may be especially difficult. The distinction between a mild psychiatric disorder and normal variation in behavior can be difficult, and in such cases the GP evaluated his/her own competence as insufficient, indicating that experts should be involved. However, the experts were regarded as complimentary, not as a reason for the GP to turn over the application process entirely to experts:

*When it comes to neurotic and personality disturbances – this is another situation, and it could be suitable to insist upon the independent evaluations of two or three specialists – psychiatrists or psychologists*.

(Norwegian GP)

##### Balancing the medical and functional/social criteria

The medical criteria were underlined by the Norwegian participants as important but other factors such as the outcome of the rehabilitation process and the social situation were evaluated on at least the same level.

*The medical arguments are only arguments which should be taken in account. We need to focus on them... Sometimes, however, especially with regard to psychiatric patients, the social situation and the possibility to get a job can strongly influence the decision*...

Even representatives of the medical profession, GPs and psychiatrists, underlined that the rehabilitation criterion was the most important. This is expressed below:

*What exactly they have tried is difficult to know. You can read that they have tried all that is needed. First of all, I would like to know if they have tried vocational rehabilitation ... or if he has been evaluated at an employment office*.

(Norwegian GP)

The SIO officer agreed about the importance of the medical criteria, but they showed broader interest into the background information about the patient. They wanted to have a total picture of the patient and an awareness of the entire situation before making a final decision. As is clear from the next quotation, the background information is rutinely collected not only from the patient, but also in other ways.

*The medical certification is necessary in all cases. We have also to know about their background, working experience... The patient self report on these questions, as usual, and we have also information registered in our data system. So we have report about all of them where they have worked, income etc*...

(Norwegian Country SIO officer)

The Russian participants evaluated the medical criterion as the most decisive one and pointed out that the other factors were usually consequences of the medical condition. They were also interested in patient's working experience and social situation, but this was evaluated as additional information that illustrated the severity of the disorder.

*We would base our decision on clinical data here. It is quite clear that he has some paranoid symptoms which hinder him not only at work, but also socially. It is also very important that he doesn't have insight into his condition*.

(Russian psychiatrist, mental hospital)

Not only medical doctors, but also social workers with not-medical professional background placed the criteria in the same order:

*I think that first of all, his health condition is important. And other factors... If he can not make money, it is mostly consequence of his health condition which is reason for all the other factors... Delusions! He has never managed to get rid of them. He can not concentrate on a concrete task*.

(Russian social worker, mental hospital)

Another tendency was that the psychiatrist working at the out-patient clinic was more prone to put weight on social factors and introduced the phrase "*social maladjustment*" as the most important aspect. Thus, it appears that the site of the evaluation (mental hospital versus out-patient clinic) may influence the weighting of the criteria more than the professional background of the persons involved.

*The degree of social maladjustment is the most importent criterion. When the patient is in such a poor clinical state that he can not manage to be adapt to society. That is the reason he has not been able to work so long time*.

(Russian psychiatrist, out-patient department)

The criteria for evaluation of a functional reduction are more detailed in Russia, where the criteria have been operationalised (see below). The Russian psychiatrists have the guidelines for the evaluation present on their desks, indicating that the criteria are well known by the professional community.

*We have a table with detailed explanation of all criteria on our desk... Every medical doctor working here should have such table*...

(Russian psychiatrist, mental out-patient department)

### 3. Rules governing appeals in disability pension cases

In Norway, an appeal can be made to the administrative body that made the decision. If the first decision is upheld, there is a possibility of making an appeal to a Social security court. The court received more than 4000 cases during 2006 in all fields of social security [30]. Appeals in disability cases are the largest group, almost 1300. In about 75 percent of the cases the original decisions were confirmed, in the rest the decisions were changed to the benefit of the applicant or returned to the local social security office for more information.

In Russia, the patients can appeal to the "Main Bureau of Medical-Social Expertise" (MBMSE) which is superior of the local bureau that made the decision. If the first decision is upheld, the next appeal can be sent to the Federal Bureau of Medical-Social Expertise (FBMSE). There is also the possibility of making an appeal to a Social security court. It was not possible to find comparable statistical data about the number of appeals in disability cases on the Russian side.

### 4. The magnitude of the DP grants

In Norway, disability pension consists of two parts: basic pension and the additional pension. The basic pension is based upon the basic amount (BA) which is regulated by the Norwegian Parliament every year on the background of changes in the income level in the country. From May 2005 the BA has been 60699 NOK (about 9,500 USD). The additional pension is estimated from the income level the patient has had during the last three years before the disability ensued. A patient who has not been employed and never had an income, gets a special supplement the size *of *which is determined yearly by the Norwegian Parliament. The size of the DP depends also on the percentage of reduction in earning ability which can be graduated from 50% to 100% with intervals in 5% (§ 12-7) [22]. However, the minimal size of annual benefit is not less than 1. 8 BA (§ 12–13). The average DP in 2004 was about 139000 NOK (about 21,000 USD) which approx. was 41% of average salary (336000 NOK = 52390 USD). Compared to the official poverty line (PL) which is 50% of the median-income for Norway [31], which was 88000 NOK (13720 USD) in 2005, the average DP was almost double.

In case of the patient who has not been granted a DP, or if the granted DP does not cover his/her basic needs, he/she has right to apply for the social support to cover his/her subsistence expenditures. The average social support for each recipient was 7118 NK (about 1,100 USD) per month in 2004. Housing expenditures may also be covered through an additional grant [32].

In Russia, DPs also consist of a basic part and an additional part. The size of the basic part is fixed every year at a set rate for different categories of beneficiaries and depends also upon number of disabled members of his/her family. The additional part depends upon the income level during the period of time before start of the disability period, the length of service and the insurance premiums. The size of the basic part of the DP also depends upon which of the disability group (DG) the patient belongs to. DGs are defined on the background of the degree of functional reduction with specific criteria for each group. The first DG – the most severe one – consists of the patients who have had an extensive reduction in their general functional disability which resulted in severe social dysfunction that requires constant help and supervision. The second DG consists of those patients with extensive reduction in general functional disability who can take care of themselves and to some extent, participate in work if the working situation is adjusted to their capacity. The third DG consists of patients with less extensive functional reductions, who need to change their work to one requiring a lower level of qualification and that is less remunerative.

DP can be given as a "social pension" (SP) for persons who have never been employed. The size of the "social pension" can be compared with the size of retirement pension: pension of the first DG is 50% of minimal retirement pension (MRP), pension of the second DG, 100% of MRP and pension of the first DG, 200% of MRP.

The average pension (both DP and retirement pension) in Russia was 2569 Rubles (103 USD) per month in 2005. This corresponds to approximately 31% of the average salary the same period of time (8299 Rubles = 331 USD). These numbers can be compared to the "minimum subsistence level" (MSL) calculated on the background of minimal need for foods, goods and services. This indicator is determined in each county four times each year, and is used to measure quality of life, for determining social and other benefits, preparation and realization of social programs and developing of budgets concerning social political issues. The average MSL in Russia was 2738 Rubles (109 USD) in the last part of 2005, which means that the average pension was a little less than the MSL. Several of our respondents remarked on the insufficient size of DP in terms of covering a realistic MSL. Below is one example of this:

*Social benefits are so small... including the disability pension... it is not the point if one gets 600 or 1500 rubles per month. All they are under the survival level... no one could live on this amount alone*.

(Russian psychiatrist, mental hospital)

## Discussion

This study has revealed noteworthy differences in the organisational model and process of applying for DP and its granting in Russia and Norway. In Norway the DP application process is initiated much later following a comprehensive occupational rehabilitation together with adequate treatment a minimum of 3 years before a DP is granted. In Russia, the application procedure starts early if the prognosis is considered unfavourable. There are two instances responsible for preparing the medical certification for a DP in Russia compared to one in Norway. Decisions are taken collectively in Russia while in Norway the PD and SIO offiser often make the decision alone. In Russia, the medical criterion is the most decisive one, while treatment and rehabilitation criteria are more central to the medical criteria in Norway. The size of DP in Norway is enough to cover of subsistence expenditures, while the Russian DP is below the level of minimum subsistence level.

### Strengths and limitations of the study

Social insurance schemes in the western countries are different in many aspects, and there are still very few comparative reviews addressing to this issue [14,33]. It means that the question if the Norwegian social insurance system is representative for the whole of Western Europe is difficult to answer. Generally, the Norwegian system is considered strongly influenced by the state and is highly universal; all citizens do under certain circumstances (sickness, disability) have the same rights to benefits. The Norwegian system has also been characterized as having a large scope of public social policy, offering a large numbers of different types of support [13,34].

Systematic cross-cultural research is important, as it allows for the comparison of strengths and weaknesses in different systems. One should bear in mind that this study is the first study comparing the Norwegian DP system with the Russian one. The aim of this first study is to give a broad overview of the systems. The details of their legislative base and how the systems are actually used in practice are not extensively covered. In addition, the qualitative interviews must be considered hypothesis generating. However, those representing all stages in the application process were interviewed in both countries. The composition of the research group encompassed both Russian and Norwegian linguistic and cultural competence. All participants were interviewed in their native tongue in order to avoid language difficulties.

### The concept of disability

The conception of "disability" has somewhat different connotations in the two countries. In Norway, a chronic psychiatric disease is more assosiated with "rehabilitation" and "economic support". A patient is not considered disabled until thorough treatment and rehabilitation has been attempted. In Russia, the connotations are "treatment" and "disability". The word invalid (disabled person) is in general use both in the Russian legislative system as well among people in general. Its extensive use can lead to a decrease in social status and stigmatization. Other studies indicate that extensive ordinary use of the word "invalid" might be one of the reasons why so few patients with neuroses and personal disorders apply for DP in Russia [35]. If one can chose, one choses not to be labelled as "an invalid". Our study also indicates that the concept of disability covers a broader range of diagnoses in Norway where neurosis and personality disorders can be reasons for granting a DP. In addition, in Norway, the functional criteria seem to be more included into the concept of "disability" than in Russia.

### The DP granting process

#### Initiation of the process and medical evaluation

The major difference between the two countries on the time point, when the application for DP process is initiated, is a factor that has great impact upon the DP granting process. In Norway, the doctor has a 3 year observation period with extensive treatment and rehabilitation information about chronicity and severity of the disorder, when the medical certificate – the basic document in the evaluation process – is completed. The Russian doctors determine a tentative prognosis based upon only a short observation period. This gives the Norwegian doctor the possibility to take into account considerably more information about the patient whereas their Russian counterparts have to rely upon their general clinical experience with similar patients to make predictions about the prognosis. Most of the evaluation probably relies upon the specific diagnosis, which is likely why the Russians put more emphasis upon the quality of the diagnostic work. Thus, it makes sense that the Russian medical assessment has to be made by a group of psychiatrists while the assessment is made by one doctor in Norway makes sense. However, the Norwegian model seems to be very sensitive to individual judgements and the possibility for individual variations. The well described conflict between the loyalties to the individual patient versus the loyalty to the society as whole when single doctors are the gate-keepers [36,37] is probably connected to the important role the medical doctors play in Norway. The collective decision made by the group of three medical doctors, when two of them are not in a direct therapeutic relationship with the patient, may lead to a more balanced evaluation and reduce the loyalty conflict and may counteract any misuse of the system. On the other hand, the Russian model, with several steps and more experts involved in the evaluation and decision-making process and the lack of objective criteria for mental disorders likely represents an additional filter for to the granting of a DP. This may explain why patients with less severe mental disorders are less likely to get a DP in Russia.

#### The size of the DP

The size of the disability pensions in both Norway and Russia is significantly below the average salary for persons at work, but in comparison between the two countries, the DP in Russia is not high enough to cover the basic need for the patient. The assumption by the Russian clinicians that the severe ill patients can not be discharged from the hospital because they cannot cope economically is warranted. We consider this fact to be a major obstacle for deinstitutionalization processes in Russia both influencing discharges from the hospitals as well as from internats for chronic psychiatric patients.

## Conclusion

Application for DP in Norway is designed as a step following an unsuccessful comprehensive treatment and rehabilitation period. In Russia, application for DP starts very early in the course of the disorder and encompasses the rehabilitation period. Our hypothesis is that this is one of the consequences of the lack of other stabile financial support for patients with long term illnesses in Russia.

The medical doctors have a dominant position in all steps of the collective evaluation and decision-making processes in Russia, while the Norwegian medical doctors have the responsibility only for medical certification and have less direct influence on the final decision. Our hypothesis is that this reflects the relative strong position of the medical doctors in the Russian health care system based upon tradition and culture.

The concept of disability is broader in Norway than in Russia. In Norway, the concept includes a broader spectrum of disorders and focuses upon social and functional criteria to a large extent than in Russia. Our hypothesis is that this is the explanation for the fact that patients with severe disorders dominate among psychiatric patients receiving DP in Russia.

The wide use of the word "invalid" in Russia may lead to a higher level of stigma attached to a DP application. That can be one of the reasons why the patients with milder psychiatric disorders avoid applying. The medical criteria are undoubtedly emphasised as the most important criterion for granting DP in Russia. In Norway, the medical criterion has less importance. The 3 year mandatory rehabilitation period is actually more decisive in the granting of a DP.

DP alone is not enough to live on in Russia, and is not attractive for the patients with milder psychiatric diagnoses who have the possibility taking care of him/her self and his/her own family. The Norwegian DP is large enough to live on and can in this sense, be regarded as an attractive goal.

## Competing interests

The author(s) declare that they have no competing interests.

## Authors' contributions

GR participated in designing the study, was responsible for data collection, participated in data analysis and interpretation of data, and was responsible for writing of the manuscript. WS participated in designing the study, data collection, data analysis and interpretation of the data, revision of the manuscript. TØ participated in data analysis and revision of the manuscript. RO participated in designing the study, data analysis and interpretation of data, was responsible for revision and writing of the manuscript. GM was responsible for designing the study, participated in data collection, and was responsible for data analysis and interpretation of data, revision and writing of the manuscript.

## Pre-publication history

The pre-publication history for this paper can be accessed here:


